# Integrating scRNA-seq to explore novel macrophage infiltration-associated biomarkers for diagnosis of heart failure

**DOI:** 10.1186/s12872-023-03593-1

**Published:** 2023-11-16

**Authors:** Shengnan Li, Tiantian Ge, Xuan Xu, Liang Xie, Sifan Song, Runqian Li, Hao Li, Jiayi Tong

**Affiliations:** 1 Department of Cardiology, Zhongda Hospital of Southeast University, Nanjing, 210009 Jiangsu China; 2 School of Medicine, Southeast University, Nanjing, 210009 China; 3 The Laboratory Animal Research Center, Jiangsu University, Zhenjiang, 212013 China

**Keywords:** Heart failure, Immune infiltration, Machine learning, Biomarker, Macrophage

## Abstract

**Objective:**

Inflammation and immune cells are closely intertwined mechanisms that contribute to the progression of heart failure (HF). Nonetheless, there is a paucity of information regarding the distinct features of dysregulated immune cells and efficient diagnostic biomarkers linked with HF. This study aims to explore diagnostic biomarkers related to immune cells in HF to gain new insights into the underlying molecular mechanisms of HF and to provide novel perspectives for the detection and treatment of HF.

**Method:**

The CIBERSORT method was employed to quantify 22 types of immune cells in HF and normal subjects from publicly available GEO databases (GSE3586, GSE42955, GSE57338, and GSE79962). Machine learning methods were utilized to screen for important cell types. Single-cell RNA sequencing (GSE145154) was further utilized to identify important cell types and hub genes. WGCNA was employed to screen for immune cell-related genes and ultimately diagnostic models were constructed and evaluated. To validate these predictive results, blood samples were collected from 40 normal controls and 40 HF patients for RT-qPCR analysis. Lastly, key cell clusters were divided into high and low biomarker expression groups to identify transcription factors that may affect biomarkers.

**Results:**

The study found a noticeable difference in immune environment between HF and normal subjects. Macrophages were identified as key immune cells by machine learning. Single-cell analysis further showed that macrophages differed dramatically between HF and normal subjects. This study revealed the existence of five subsets of macrophages that have different differentiation states. Based on module genes most relevant to macrophages, macrophage differentiation-related genes (MDRGs), and DEGs in HF and normal subjects from GEO datasets, four genes (CD163, RNASE2, LYVE1, and VSIG4) were identified as valid diagnostic markers for HF. Ultimately, a diagnostic model containing two hub genes was constructed and then validated with a validation dataset and clinical samples. In addition, key transcription factors driving or maintaining the biomarkers expression programs were identified.

**Conclusion:**

The analytical results and diagnostic model of this study can assist clinicians in identifying high-risk individuals, thereby aiding in guiding treatment decisions for patients with HF.

**Supplementary Information:**

The online version contains supplementary material available at 10.1186/s12872-023-03593-1.

## Introduction

Heart failure (HF) is a multifaceted clinical syndrome that arises due to the progression of various cardiac diseases. The structural and functional abnormalities of the heart can cause impaired cardiac filling or blood ejection, leading to HF [1]. HF has emerged as a significant global public health challenge, with high rates of hospitalization and mortality, affecting approximately 24 million patients worldwide [2]. Ischemic cardiomyopathy (ICM) and dilated cardiomyopathy (DCM) are the most prevalent causes of HF. Therefore, it is crucial to take effective measures to prevent the onset of HF or explore new strategies to reduce its mortality rate.

Recent research has uncovered the role of leukocyte subclasses and various inflammatory mediators in HF and cardiovascular disease progression, with a particular focus on the interplay between immune cells such as macrophages [2, 3] and lymphocytes [4], and inflammation [5]. In previous studies on leukocytes in cardiac disease, monocytes were mainly considered as homogenous populations with a single function. However, recent research has broadened this description to encompass distinct populations monocytes, macrophages, T lymphocytes, B lymphocytes, and neutrophils, suggesting their different roles in cardiac disease. Studies have indicated that T cells have been shown to have an impact on cardiac inflammation, hypertrophy, fibrosis, and dysfunction in nonischemic HF, and macrophages contribute greatly to cardiac fibrosis and diastolic dysfunction [6]. Recently, previously unrecognized temporal and spatial roles of resident and nonresident macrophages in the progression of HF have been observed [7]. The macrophage phenotype has the potential to act as a regulator of inflammation in the progression of HF. Multiple regulators of macrophage activation have been identified and the regulation of macrophage phenotype has also been studied in the development of HF [8]. Despite these findings, the specific features of immune cells and effective molecular diagnostic biomarkers for HF remain unclear. A thorough comprehension of alterations in the immune microenvironment changes of diseased hearts could be a crucial step in revealing potential therapeutic approaches.

Over the past few years, high-throughput sequencing technologies like microarray, RNA-sequencing (RNA-seq), and single-cell RNA-sequencing (scRNA-seq) have been utilized to explore immune cell distribution and identify effective diagnostic biomarkers via several gene expression profiles. Machine learning has played a crucial role in discovering vital cell types and diagnostic markers because of its efficiency in identifying relevant biomarker features, and classifying and validating biomarkers [9, 10]. However, there is still a lack of comprehensive characterization of immune cell components and their influence on HF. This study used the CIBERSORT method to calculate the quantity of 22 immune cells, identified essential cell types using machine learning algorithms, screened hub genes associated with key cell types, and validated hub genes in clinical patients. The objectives of our study were to explore the critical roles of immune cells and genes in the pathogenesis and advancement of HF to provide fresh insights for disease diagnosis, treatment, and understanding of immunity.

## Methods

### Data collection

**Fig. 1:**
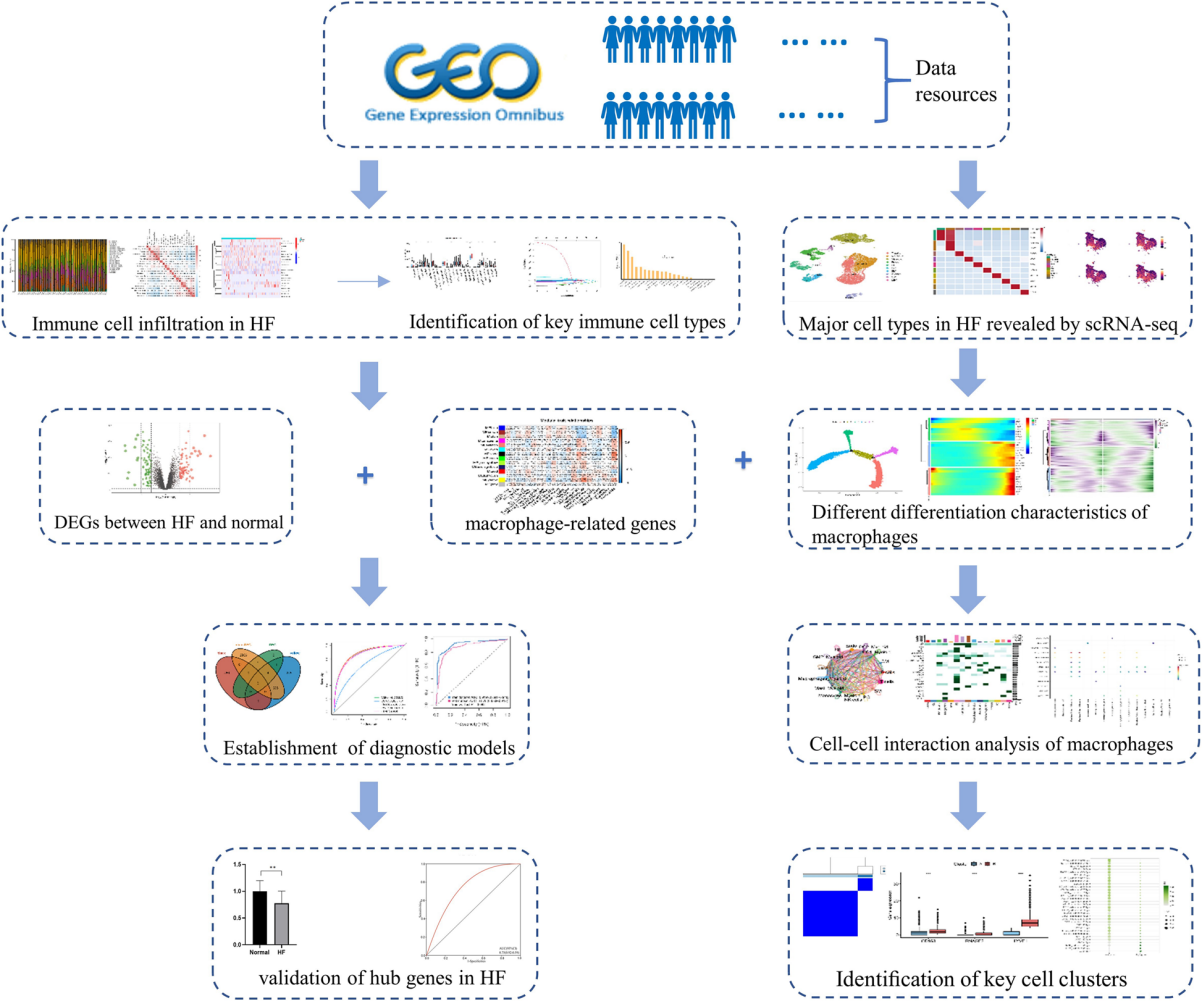
Research design flow chart

The research flowchart is presented in Fig. 1. The gene expression profiles of HF in the training set and the validation set were obtained from GEO database (http://www.ncbi.nlm.nih.gov/geo). The training set included GSE3586 [11], GSE42955 [12], GSE57338 [13] and GSE79962 [14], and the validation set was GSE116250 [15]. Single-cell data of HF was downloaded from GSE145154 [8].

### Data pre-processing

The R package GEOquery was utilized to download GSE3586, GSE42955, GSE57338, GSE79962 and GSE116250. The gene expression matrix was converted according to the platform annotation file. The expression matrix of the array data was normalized using robust multichip average. If more than one probe corresponded to one gene, the average was taken. Subsequently, we combined the four datasets (GSE3586, GSE42955, GSE57338, GSE79962) into one training dataset and employed the “ComBat” in sva package to eliminate batch effects. We employed principal component analysis (PCA) for the visualization of the data and to detect any potential batch effects in the merged dataset.

### Immune cell infiltration analysis

To study the disease immune microenvironment, we utilized the R package “CIBERSORT” to calculate immune cell infiltration based on the standardized gene expression data. The results of immune cell infiltration were displayed using the ggplot2 and pheatmap packages. We performed Spearman analysis and visualized the results of immune cell infiltration correlation using the corrplot package. Furthermore, a range of methods was applied to assess immune cell infiltration as well, including “xCell”, “MCPcounter”, “ssGSEA” and “ABIS”.

### Least absolute shrinkage and selection operator (LASSO) regression and random forest analysis

We constructed a LASSO prediction model utilizing the “cv. glmnet” function in the glmnet package. The parameters (alpha = 1 and nlambda = 1000) were set in the analysis and lambda. Min was selected as the optimal lambda. Additionally, we utilized “RandomForest” function to conduct a random forest analysis [16]. To determine the importance of the indices, we calculated the percentage increases in the mean squared error (MSE) of each variable, with higher MSE% values indicating more important variables [17]. The key differential immune cells were then screened using the differential immune cells obtained from the random forest analysis and the LASSO regression.

### Differentially expressed genes (DEGs) analysis

We conducted DEG analysis using the R package “limma” with a threshold of |log2 fold change (FC)| > 1 and false discovery rate (FDR) < 0.05 [18]. The volcano plot was displayed to visualize the results.

### Functional enrichment analysis of DEGs

Using the ClusterProfiler package, we conducted GO functional enrichment analyses. The GO analysis included three main components: biological process, cellular component, and molecular function. Statistical significance was defined as adjusted *p* values less than 0.05.

### Dimensionality reduction, clustering, visualization, and cell type recognition

Seurat was utilized to reprocess the data and annotate cell clusters. PCA was applied to reduce the dimensionality of integrated data. Using the first 10 principal components (PCs), we further reduced the integrated dataset to a two-dimensional space and visualized it by UMAP. According to the marker genes [8, 19], the 58,233 cells were annotated as Endocardium (Endo), Fibroblast (FB), Pericytes (PC)**,** Smooth muscle cells (SM), Cardiomyocytes (CM), Lymphatic endothelial cells (LEC), Myeloid, T cells, NK cells, and B cells.

### AUCell gene set enrichment analysis

The AUCell package calculates AUCell scores to mark genes characteristic of each cell within macrophages and display interactive UMAP maps of the resulting scores. The gene sets “h.all.v7.1.symbols.gmt” were from the MSigDB database.

### Cell-cell interaction analysis

To enable a systematic analysis of cell signaling pathway communication, the “CellChat” package was adopted. We visualized the interaction between different cell subpopulations through putative ligand-receptor pairs using the ggplot2 package and “Webr” package (version 0.1.5).

### Analysis of single-cell trajectories

Pseudotime trajectories of macrophages were explored by the Monocle (v2.22.0). The package employed machine learning techniques to arrange cells into trajectories with branch points based on a specific set of genes as input. The findings indicated that different clades corresponded to cellular populations with unique differentiation states. Differential analysis was performed between branches, and these macrophage marker genes located in different branch states were defined as macrophage differentiation-related genes (MDRGs).

### WGCNA

We analyzed the immune-related genes and gene modules through WGCNA (weighted gene coexpression network analysis) using the R package ‘WGCNA’ [20]. A suitable soft threshold of 10 was selected using the Pick Soft Threshold function, and 14 modules were established through dynamic branch cutting with 0.25 as the merging threshold.

### Patients and variables

The information of samples was collected from July 2022 to April 2023. A total of 40 patients with HF and 40 controls without HF in hospitalized patients during the same period were enrolled consecutively from Zhongda Hospital of Southeast University (Nanjing, Jiangsu, China). Disease was diagnosed based on a patient’s medical history, clinical performance, auxiliary examination, and case notes by specialized expert cardiologists. Patients were required to meet several criteria, including evidence of structural heart disease and manifestation of circulating congestion, age equal to or greater than 18 years old, New York Heart Association (NYHA) class equal to or greater than II, a minimum N-terminal pro-B-type natriuretic peptide (NT-proBNP) level of 400 pg/mL, and willingness to provide written informed consent. In addition, HF can also be diagnosed when patients have significant signs and symptoms of HF described above, but NT-proBNP levels are less than 400 pg/mL if their left ventricular ejection fraction (LVEF) is less than 40%. Patients who had been hospitalized for HF within the previous 12 months needed to have a NT-proBNP concentration of at least 600 pg/mL, while those with atrial fibrillation or atrial flutter required a level of at least 900 pg/mL, regardless of their history of HF hospitalization. Several exclusion criteria were applied in our study, which comprised recent worsening HF or other cardiovascular events or procedures, estimated glomerular filtration rate (eGFR) below 30 mL/min/1.73 m^2, acute or previous myocardial infarction, as well as moderate-to-severe liver and kidney dysfunction. Details of the patients are provided in Supplementary Table 1 and Supplementary Table 2. This study was approved by the Ethical Committee of Zhongda Hospital of Southeast University in Nanjing under the number 2021ZDSYLL111-P01. Written informed consent was obtained from all patients, and the experiments were performed in accordance with the approved study protocol.

### Quantitative real-time polymerase chain reaction (qRT–PCR)

Total RNA was extracted from peripheral blood of HF patients and healthy people utilizing the RNAprep Pure high efficiency total RNA extraction kit (TIANGEN, China). A cDNA synthesis kit (R323, Vazyme Biotech co., Ltd) was used to reverse transcribe the extracted RNA, and SYBR qPCR Master Mix (High Rox, Q341, Vazyme Biotech co., Ltd) was used for quantitative PCR of 2 diagnostic genes. The 2-ΔΔCt method was applied to estimate the relative expression of the target genes. GAPDH was used as an internal control, and the primers are listed in Supplementary Table 3.

### Regulatory analysis of transcription factors (TFs)

Unsupervised clustering analysis was used to categorize macrophages into different patterns. Based on the consensus clustering algorithm, the number of clusters and their stability were determined [21]. The Consensus Cluster Plus package was applied to run the above steps and was repeated 1000 times to secure the stability of the results. To infer TF-target interactions in the cluster with different biomarker expressions, “SCENIC” [22] package was used. On the basis of co-expression network, SCENIC recognized potential TF targets and identified direct targets (regulatory factors) by TF motif enrichment analysis and calculated the activity of regulators on single cell. Scatter plots were used to illustrate the TFs with regulation specificity scores. Additionally, the specificity scores of the top five TFs that only existed in the low expression group were further analyzed. Expression comparison between the groups was conducted to explore the TFs that may influence the expressions of biomarkers.

### Statistical analysis

Statistical analyses were mainly performed using R (version 4.1.2) and GraphPad Prism (version 8.0.1). Data were expressed as median (interquartile range) or mean (± standard error of mean, SEM). For comparisons of continuous variables between two groups, normally distributed variables were evaluated using independent Student’s t-tests, and non-normally distributed data were analyzed using Mann–Whitney U tests (the Wilcoxon rank sum test). Kruskal-Wallis test was performed when analyzing more than two groups. The relationships between gene expression levels were evaluated on the basis of Spearman correlation coefficients. Receiver operating characteristic curves were plotted using the SurvivalROC package, and the area under the curve was used to evaluate the accuracy of the gene signature. If not specified, *P* < 0.05 was considered statistically significant.

## Result

### Immune cell infiltration in HF

**Fig. 2:**
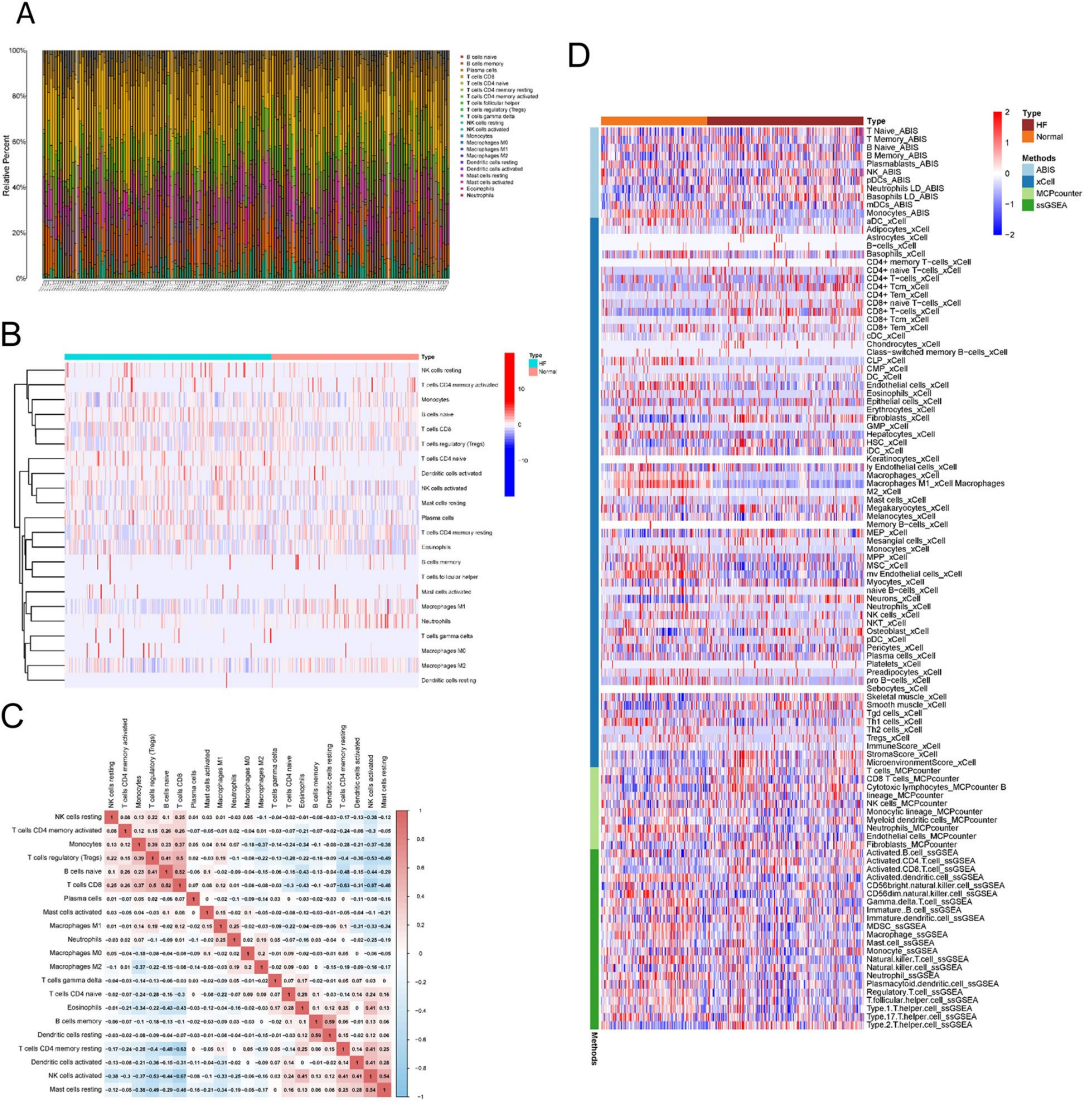
Immune cell infiltration in HF. (**A**) Bar plot showing the composition of 22 types of immune cells across samples. (**B**) Heatmap of the composition of 22 types of immune cells across samples, colored by normalized relative abundance. (**C**) Correlation heatmap of 22 types immune cells in HF samples. Red indicates positive correlation, and blue indicates negative correlation. (**D**) The immune infiltration differences between HF and normal by ABIS, xCell, MCPcounter and ssGSEA methods

We used four datasets, consisting of 116 cases of DCM and 118 cases of ICM, as the training dataset for our analysis of immune cell infiltration and DEG analysis. The pertinent details of chosen datasets are presented in Table 1. After gene expression profiling and PCA, baseline batch differences were observed in the merged datasets. To increase analysis power, we applied the “ComBat” algorithm to correct for batch effect. By implementing the batch-correction methods, we were able to mitigate the batch effects to a considerable extent (Supplementary Fig. 1A). To estimate the abundance of infiltrating immune cells in HF and normal samples, we employed CIBERSORT on the corrected expression matrix (comprising four datasets). According to the results, the predominant immune cells that infiltrated in HF were macrophages, neutrophils, CD8^+^ T cells, regulatory T cells (Tregs), and naive B cells (Fig. 2A). M2 macrophages, neutrophils, and CD8 + T cells were significantly different between HF and normal (Fig. 2B). We further delved into the correlation between immune cells in HF. The results indicated that in mast cells, NK cells, and CD4+ memory T cells, the proportion of activated population was negatively correlated with that of the corresponding resting population (Fig. 2C). Additionally, we found a negative correlation between the proportion of M1 macrophages and that of resting mast cells and activated NK cells, and a positive correlation between the proportion of M1 macrophages and that of Tregs and activated mast cells (Fig. 2C). We also used four additional methods (ABIS, MCPcounter, xCell, ssGSEA) to demonstrate the immune infiltration differences between HF and normal (Fig. 2D). Notably, differences in immune infiltration of monocytes, neutrophils and NK cells were found between HF and normal by four different algorithms. In conclusion, immune environment seemed to be of great importance for the occurrence and progression of HF.

**Table 1:** Characteristics of the five datasets

Datasets	DCM	ICM	Normal	Platform	
GSE3586	13	0	15	GPL3050	
GSE42955	12	12	5	GPL6244	
GSE57338	82	95	136	GPL11532	
GSE79962	9	11	11	GPL6244	
GSE116250	37	13	14	GPL16791	
GSE145154	2	2	1	GPL20795

### Identification of key immune cell types associated with HF

**Fig. 3:**
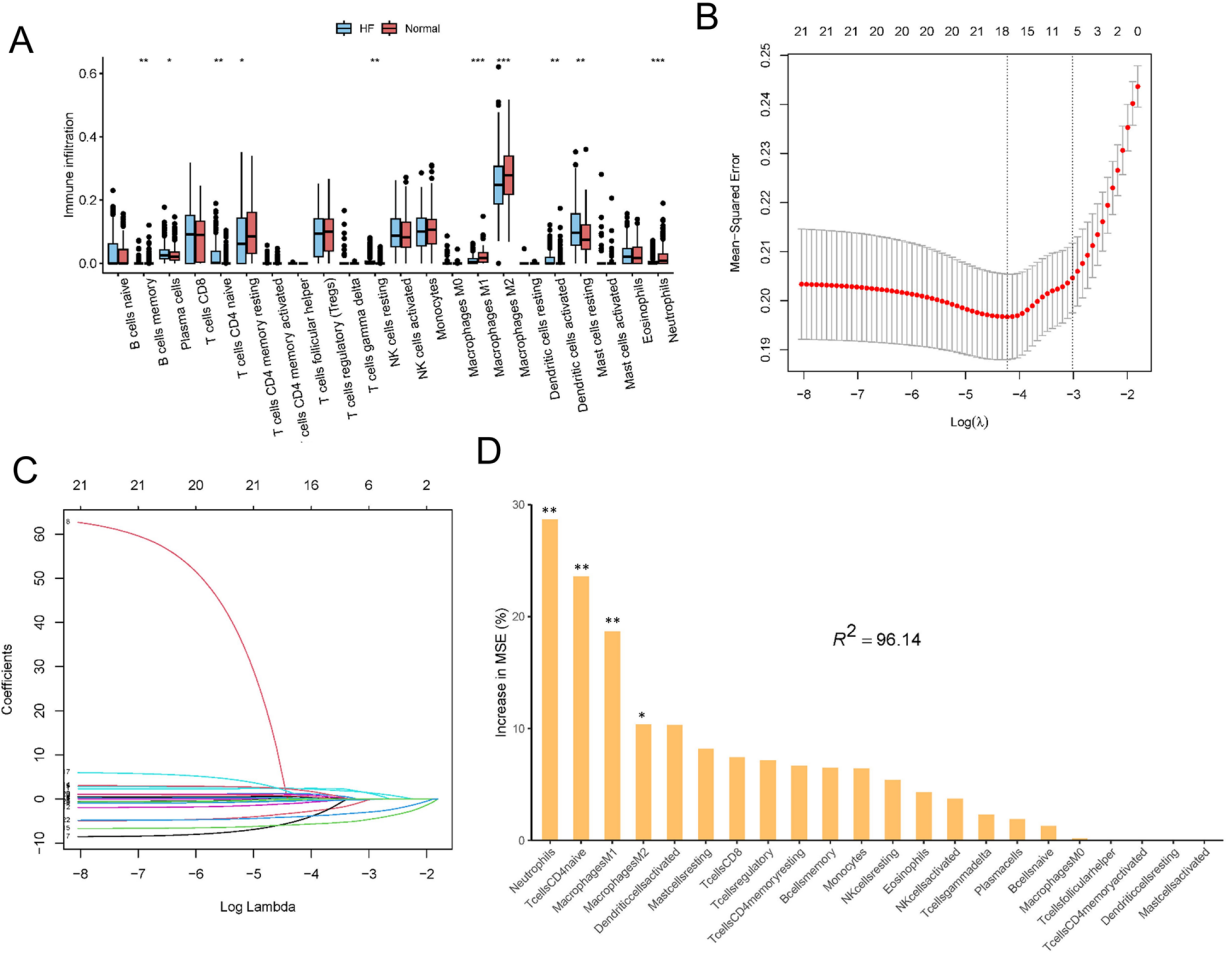
Identification of key immune cell types associated with HF. (**A**) Identifying the significantly different infiltrates of immune cells in HF and normal by Wilcoxon test. The upper and lower ends of boxes represent the interquartile range. Lines in the boxes represent median values, and dots show outliers. Statistical analysis was performed using Wilcoxon rank sum test. Asterisks indicate significance, *p < 0.05; **p < 0.01; ***p < 0.001; ns, no statistical significance. LASSO regression (**BC**) and RandomForest (**D**) were conducted to analyze the different infiltrates of immune cells in HF, *p < 0.05; **p < 0.01

Subsequently, Wilcoxon test was selected to determine the differential abundance of immune cells between HF and normal samples in the merged dataset (Fig. 3A). We found 10 immune cell types that exhibited significant differences between HF and normal. For instance, the proportion of M1 and M2 macrophages was significantly different between HF and normal, and the proportion of resting NK cells and native CD4 T cells in normal was lower than that in HF (Fig. 3A). To identify critical disease-associated immune cell types, six immune cell types associated with HF were identified using LASSO regression: B cell memory, CD4 naive T cells, resting NK cells, M1&M2 macrophages, and neutrophils (Fig. 3B, C). According to the random forest algorithm, the top four immune cells (Neutrophils, CD4 naive T cells, M1&M2 macrophages) were identified as the key immune cell types based on increase in MSE (Fig. 3D). On the basis of the union of LASSO and random forest algorithms, four immune cell types were identified as closely associated with HF: neutrophils, CD4 naive T cells, M1 macrophages, and M2 macrophages (Fig. 3B-D).

### Major cell types in HF revealed by scRNA-seq

**Fig. 4:**
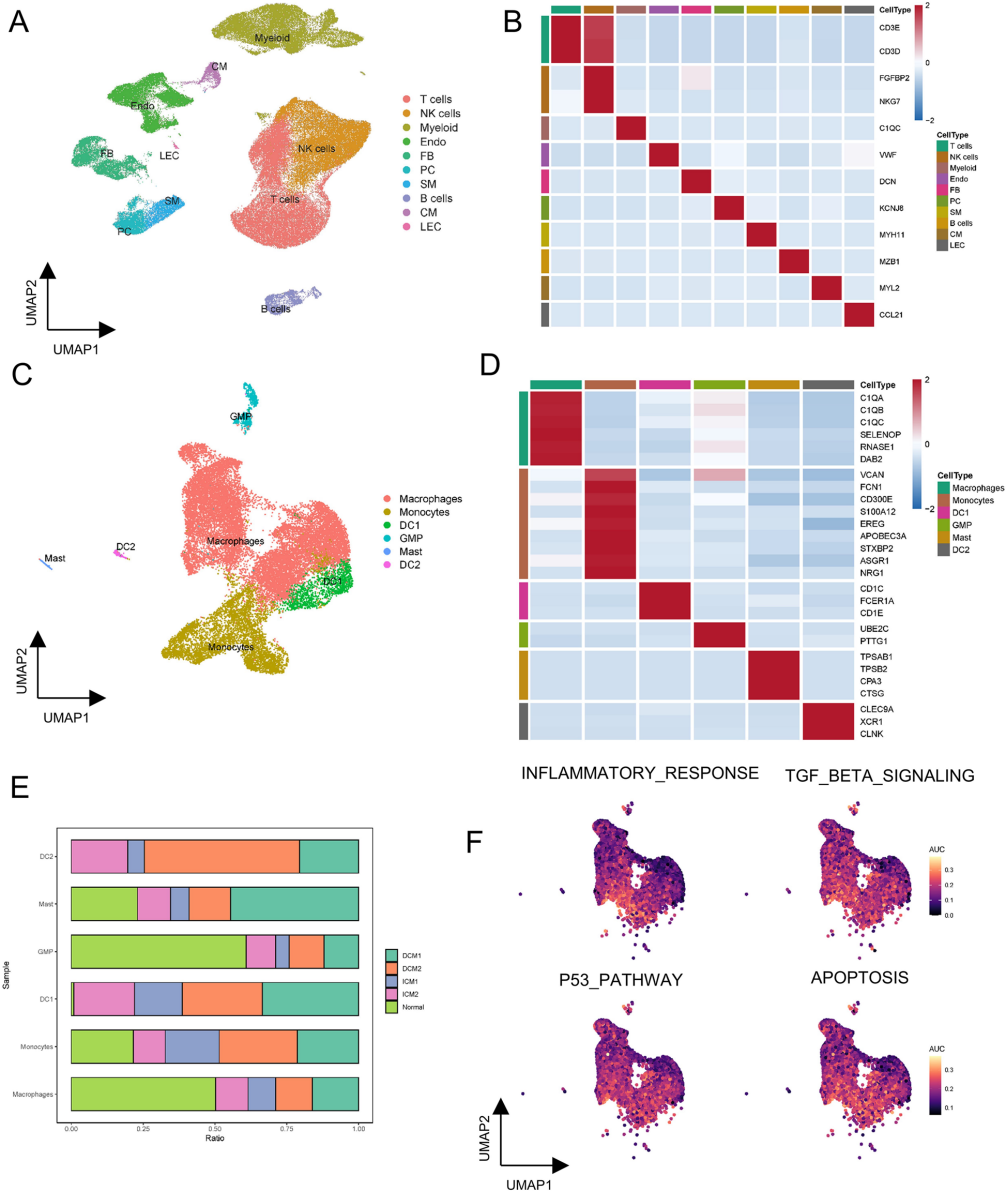
Major cell types in HF revealed by scRNA-seq. (**A**) Ten cell clusters were identified by marker gene annotation. (**B**) Heatmap of the expression level of marker genes from ten cell types. (**C**) Six cell clusters were obtained after classification of myeloid cells, and identified by marker gene annotation. (**D**) Heatmap of the expression level of marker genes from six cell types. (**E**) Bar plots showing the proportion of cell types in each sample. Statistical analysis was performed using independent Student’s t tests. Asterisks indicate significance, *p < 0.05; **p < 0.01; ***p < 0.001; ns, no statistical significance. (**F**) UMAP plots showing pathway activity for macrophages

In order to identify cell subsets expressing genes related to HF, we further collected scRNA-seq data of HF. After conducting quality control, we obtained 58,233 high-quality single-cell data. Next, we carried out normalization, unsupervised dimensionality reduction, and graph-based clustering on this dataset. We recognized a total of ten different cell subsets, including T cells, NK cells, Myeloid, Endo, FB, PC, SM, B cells, CM, and LEC (Fig. 4A). Annotations of different cell types were determined using canonical markers as well as information gathered from previously published literature [8, 19], such as CD3D and CD3E for T cells, C1QC for myeloid, and MZB1 for B cells (Fig. 4B, D). To further explore the role of macrophages in HF, we then performed a separate clustering analysis of the myeloid cell population, which revealed 6 major cell types (macrophages, monocytes, granulocyte- macrophage progenitors (GMP), dendritic cells 1&2 (DC1&2) and mast cells (Fig. 4C). We did not cluster neutrophils in this database because neutrophils are more sensitive with a half-life of only 15-20 h, which puts forward higher requirements for single cell sequencing of neutrophils. Therefore, macrophages were selected for further analysis. Figure 4E showed significant differences in the proportion of macrophages in DCM, ICM, and normal subjects. Furthermore, we found that there was a considerable AUCell score in the activity of various signaling pathways in macrophages of HF such as inflammatory response, apoptosis, p53 pathway, and TGF-β signaling (Fig. 4F). The above findings indicated that infiltration of macrophages maybe the important risk factor contributing to the process of HF.

### Cell-cell interaction analysis of macrophages

**Fig. 5:**
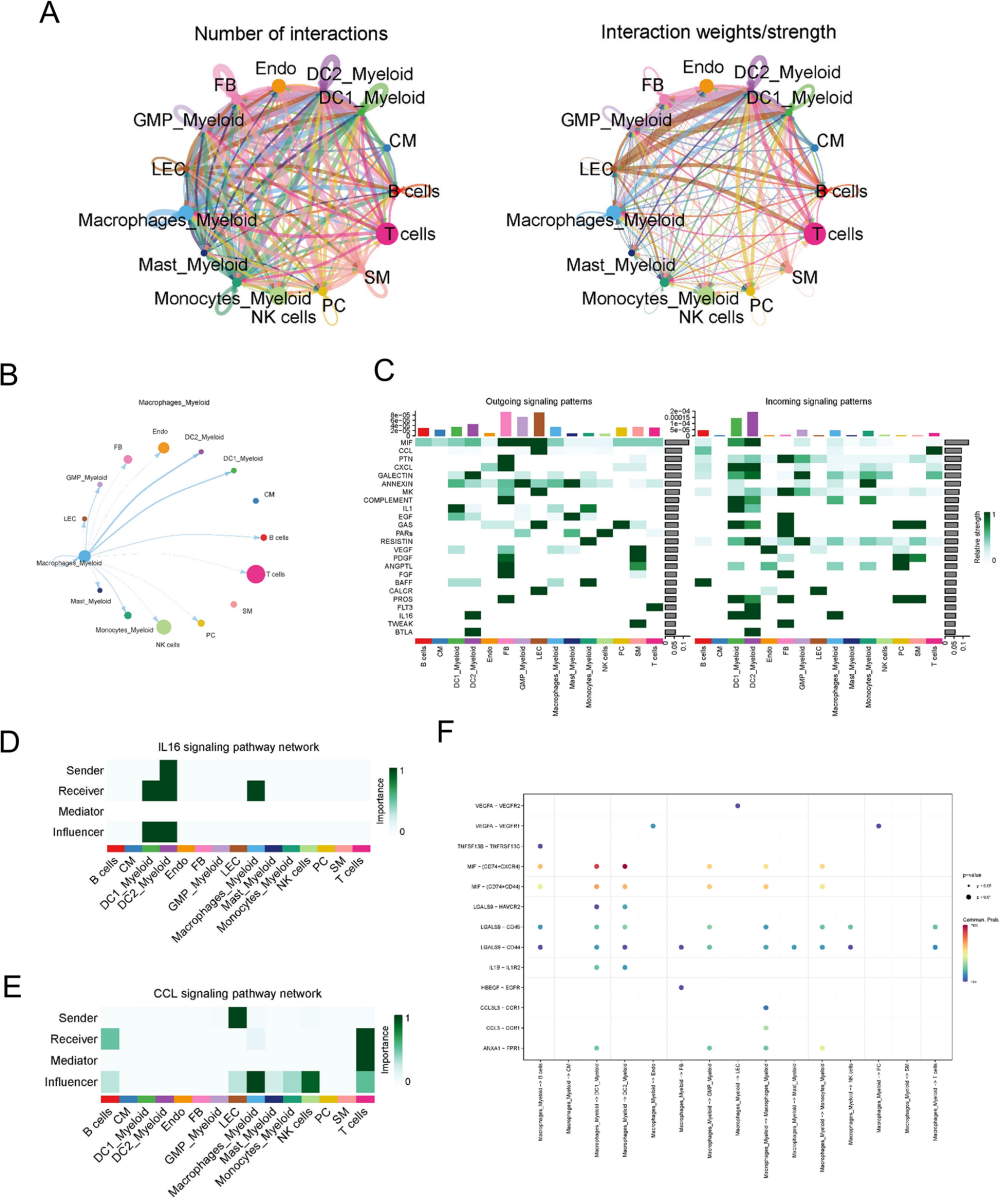
Cell-cell interaction analysis of macrophages. Circos plots showing the interactions density between any two cell types in normal (**A**) and HF(**B**). (**C**) The crucial roles of macrophages in the communication network in normal (Left) and HF(Right). (**D**) The major signaling inputs and outputs among subsets. IL-16 (**E**) and CCL (**F**) signaling pathway network and expression between all celltypes. (**G**) Bubble plot showing the ligand-receptor interactions between macrophages and other cells. *P*-values are indicated by circle size. Communication proportion is indicated by color. The redder the color, the more important the interaction

We performed cell communication analysis using CellChat to identify signal networks related to HF. Cell-cell interactions were compared in normal control (Fig. 5A) and HF samples (Fig. 5B), respectively. Interestingly, we found a denser interaction network in HF compared to normal samples. Supplementary Fig. 1B and 1C showed the overall communication conditions for all cell clusters in number and weight, respectively. To further investigate the potential influence of macrophages in HF, we explored the intercellular communication between macrophages and other cell types. We found that macrophages in HF had stronger intercellular communication with DC2 and less communication with SM than those in normal samples (Fig. 5C). CellChat detected 24 notable pathways between different clusters in HF, with the ANNEXIN signaling pathway and IL-16 signaling pathway presenting the most salient outgoing and incoming signaling patterns in macrophages (Fig. 5D). Figure 5E and F indicated that macrophages expressed the major receiver in IL-16 signaling pathway and the influencer in CCL signaling pathway. The ligand–receptor interactions that mainly involved macrophages with other cells were identified (Fig. 5G).

### Different differentiation characteristics of macrophages

**Fig. 6:**
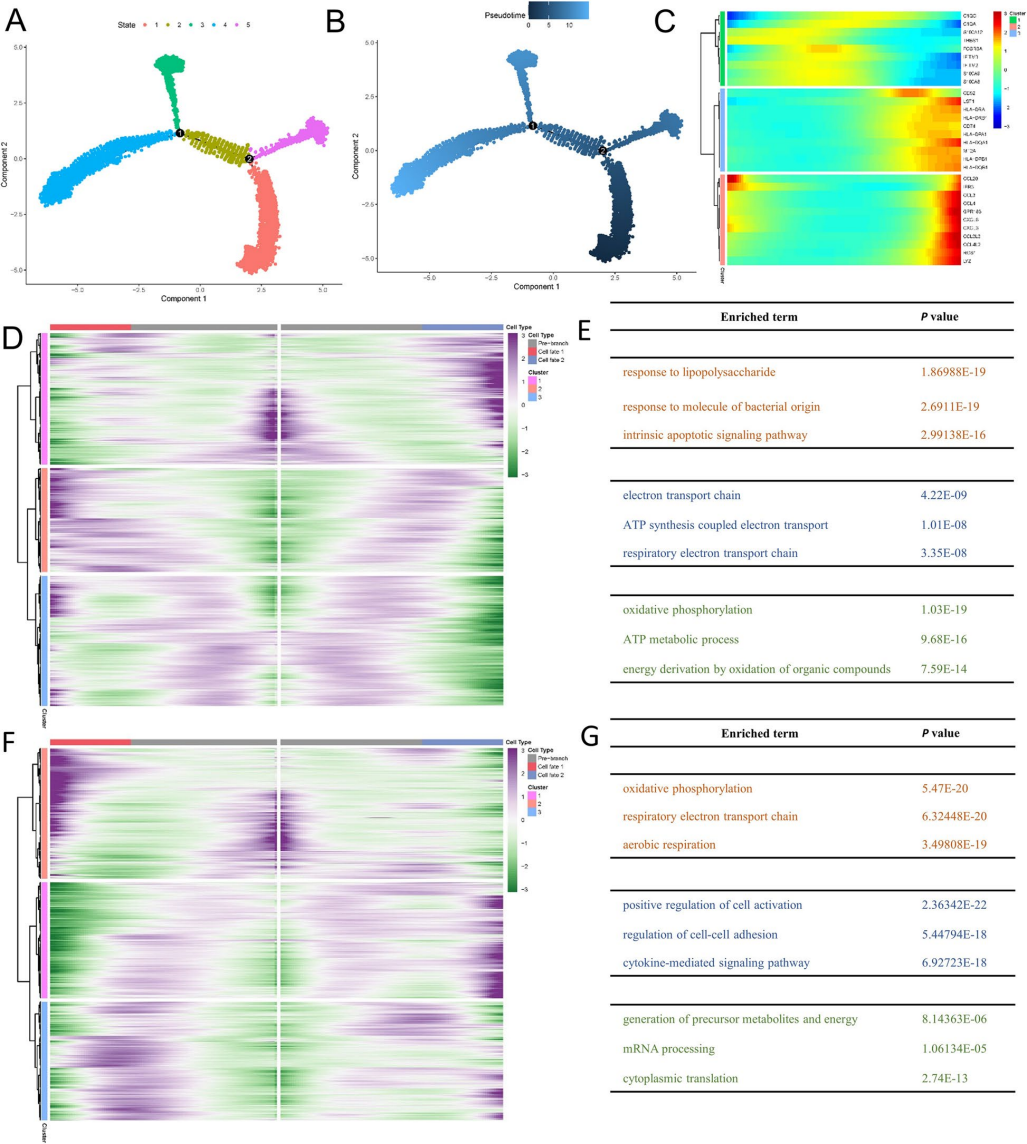
Different differentiation characteristics of macrophages. According to the pseudotime (**A**, **B**) of Macrophages, the cell population was divided into five different differentiation states. (**C**) Heatmap of top 30 differential genes. (**D**) Distribution of macrophages in different samples during the five stages. Heatmap showing the differentially expressed gene changes in branch point 1 (**E**) and branch point 2 (**G**). Go analysis of differentially expressed gene changes in branch point 1(**F**) and branch point 2 (**H**)

To understand possible developmental connections in macrophages, we performed pseudo-time trajectory analysis. Monocle 2 constructed the single-cell trajectories in pseudotime, which consisted of two branch points (five branches and five states) (Fig. 6A, B). Through differential analysis of differentiation states, we obtained Macrophage differentiation-related genes (MDRGs), and classified macrophages into three molecular subgroups (Fig. 6C). In addition, we analyzed the differences in the distribution of the different samples across the five states (Fig. 6D). The results showed that macrophages in normal subjects belonged predominantly to states 1 and 5, and macrophages in HF belonged predominantly to states 3 and 4. This suggested that macrophages progressively entered states 3 and 4 during the progression of HF and bifurcated into different cellular fates after the branch point. Figure 6E and G showed the variations in differentially expressed genes when cells in branch point 1 and branch point 2 performed different gene expression programs, respectively. Figure 6F indicated the alterations in differentially expressed genes pertinent in branch point 1. These genes were classified into three categories, which were associated with response to lipopolysaccharide, respiratory electron transport chain, and oxidative phosphorylation, respectively. Figure 6H showed the differentially expressed gene changes in branch point 2. These genes were classified into three categories involved in oxidative phosphorylation, positive regulation of cell activation, generation of precursor metabolites, and energy. Both branch points were related to multiple metabolic processes, such as respiratory electron transport chain and oxidative phosphorylation.

### Construction and verification of the prognostic risk model

**Fig. 7:**
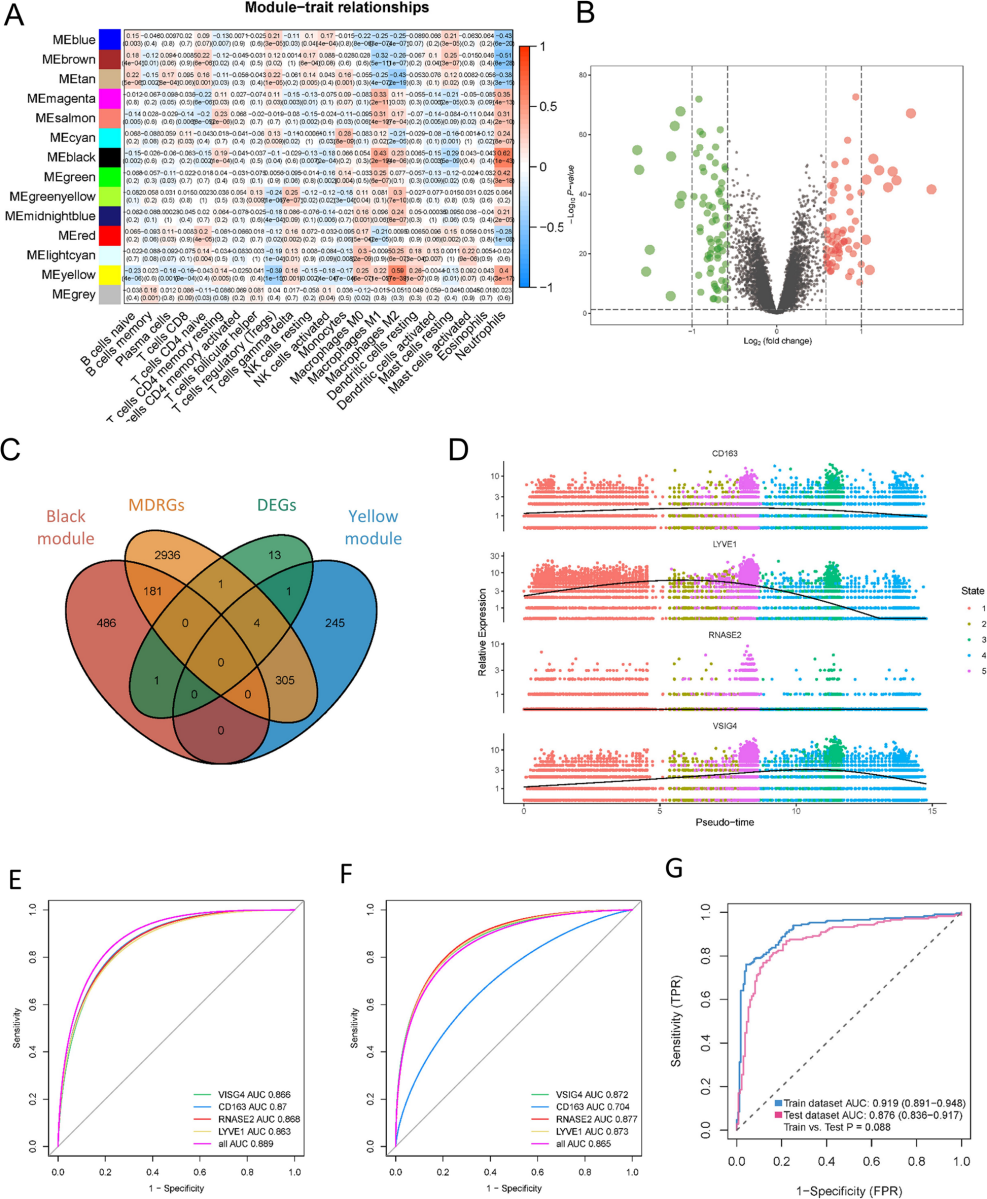
Construction and verification of the prognostic risk model. (**A**) Heatmap of the relationships between coexpression modules and immune cells. The number indicates the correlation coefficients between coexpression modules and immune cells, and the number in parentheses indicates the corresponding *p* values. (**B**) The volcano map of DEGs. Black dots represent genes that are not differentially expressed between HF and normal. Green indicates down-regulated genes, and red indicates up-regulated genes. (**C**) Venn diagram showing common genes of macrophage-related genes in the black and yellow module, MDRGs and DEGs between HF and normal samples. (**D**) Dot plot of the expression of VSIG4, CD163, RNASE2, and LYVE1 in all cells. (**E**) Gene expression of the four key genes in different differentiation states of macrophages. (**F**, **G**, **H**) Diagnostic effectiveness by ROC analysis in the training set and validation dataset. ROC curves for the all factors were constructed based on binary logistic regression

WGCNA was used to analyze the module genes most associated with macrophages. While constructing a co-expression network, we discovered that the soft thresholding power β was 10 when the fit index of scale-free topology reached 0.90 (Supplementary Fig. 1B). We determined nine modules using average linkage hierarchical clustering and the soft thresholding power (Supplementary Figure1C) and found that the genes in the yellow and black modules were most significantly associated with macrophages (Fig. 7A). Subsequently, we explored DEGs between HF and normal from the merged datasets (Fig. 7B). Based on macrophage-related genes in the black and yellow module, MDRGs, and DEGs between HF and normal samples, we found that the intersection of these results yielded 4 hub genes (VSIG4, CD163, RNASE2, LYVE1) (Fig. 7C), which were significantly downregulated in HF than in normal samples. The results of GO analysis indicated that myeloid differentiation was dramatically enriched by these macrophage-related genes (Supplementary Fig. 2A). Figure 7D demonstrated the differences in the expression of VSIG4, CD163, RNASE2, and LYVE1 in different cells. Figure 7E indicated that the expression levels of four hub genes varied in the five states of macrophages. To evaluate the sensitivity and specificity of a candidate diagnostic gene, a ROC curve analysis was constructed and the area under the ROC curve (AUC) was assessed (Fig. 7F, G). We also used logistic regression to establish an HF diagnostic model containing two hub genes (CD163 and RNASE2). According to ROC analysis, these hub genes were considerably sensitive and specific regarding diagnosing HF, with AUCs were 0.919 and 0.876 in the training and validation datasets, respectively (Fig. 7H). This supported the excellent diagnostic performance of the model.

### Validation of potential biomarkers and their correlations with clinicopathological parameters

**Fig. 8:**
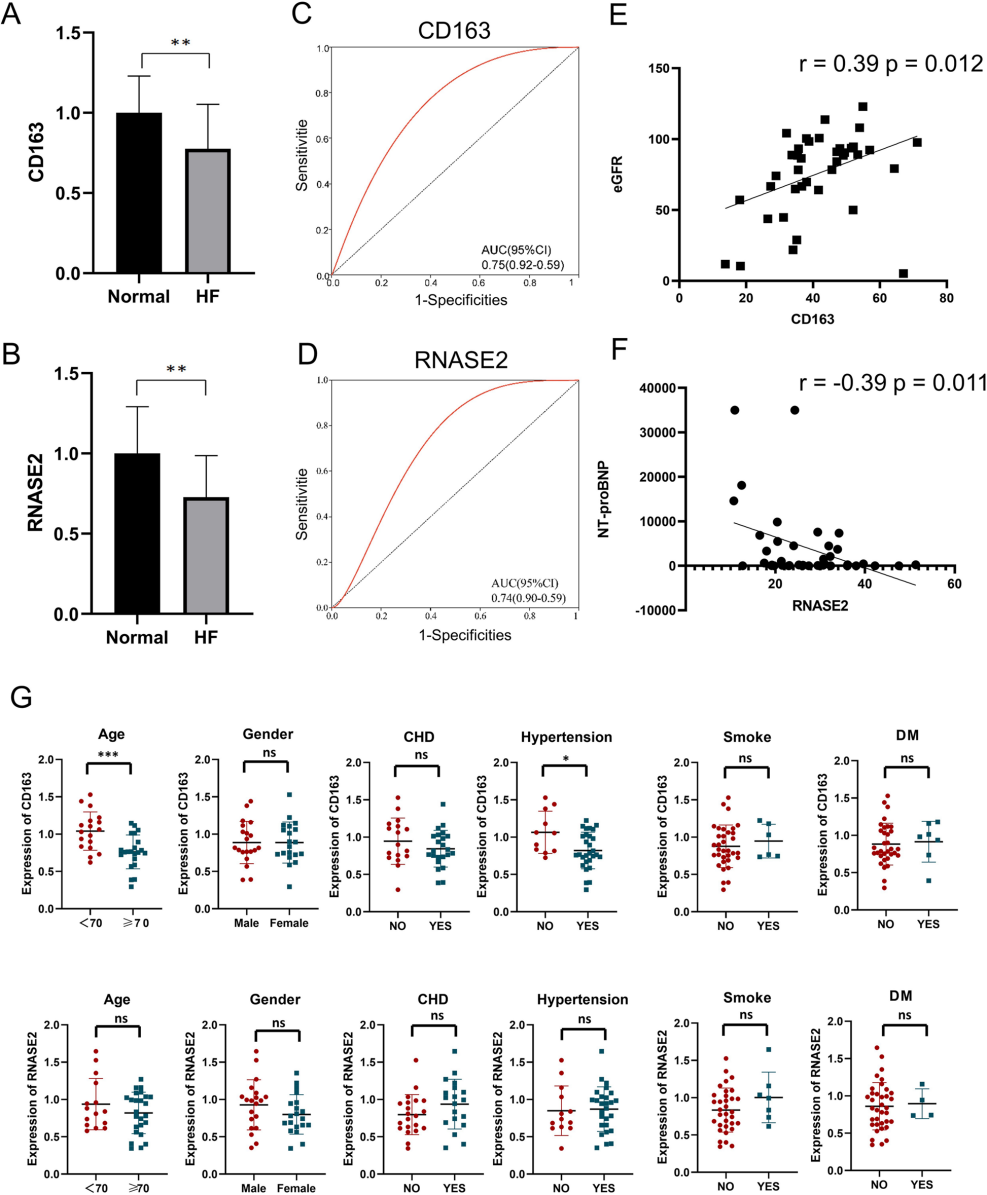
Validation of potential biomarkers and their correlations with clinicopathological parameters. The expression levels of (**A**) CD163 and (**B**) RNASE2 in normal vs. HF were analyzed by RT-qPCR. Statistical analysis was performed using the Wilcoxon rank sum test. (**C**, **D**) ROC curves of CD163 and RNASE2 for evaluating the diagnostic efficacy. (**E**) A Pearson correlation analysis of CD163 and eEGFR. (**F**) A Pearson correlation analysis of RNASE2 and NT-proBNP. (**G**) Correlation of CD163 and RNASE2 with clinicopathological characteristics. Statistical analysis was performed using the Wilcoxon rank sum test. Asterisks indicate significance, *p < 0.05; **p < 0.01; ***p < 0.001; ns, no statistical significance; HF: Heart failure; NT-proBNP: N-terminal pro-B-type natriuretic peptide; eGFR: estimated glomerular filtration rate; CAD: Coronary artery disease; DM: Diabetes mellitus

Tables 2 and 3 presented the clinical characteristics of participants in the CD163 group and RNASE2 group, respectively. The expression levels of the hub genes CD163 and RNASE2 were downregulated in HF compared to normal by quantitative RT-qPCR experiment (Fig. 8A, B). ROC curves were generated to assess the capability of these genes to distinguish HF from normal, and the AUCs of CD163 and RNASE2 were 0.75 (95% CI 0.59–0.92) and 0.74 (95% CI 0.59–0.90), respectively, indicating that CD163 and RNASE2 may serve as novel biomarkers of HF (Fig. 8C, D). Notably, the expression of CD163 demonstrated a positive correlation with the estimated glomerular filtration rate(eGFR) (*r* = 0.39, *p* = 0.012), while RNASE2 expression was negatively correlated with NT-proBNP (*r* = − 0.39, *p* = 0.011) (Fig. 8E, F). We further examined the correlation with other indicators low-density lipoprotein cholesterol (LDL-C), cardiac troponin I(cTnI), but did not find any significant correlation. We conducted an analysis of the relationships between the expression of these candidate genes and clinical pathological features, including age, sex, coronary artery disease (CAD), hypertension, smoking, and diabetes mellitus (DM), in HF patients to better understand their role in the development of HF. CD163 expression was negatively correlated with the history of hypertension (*p* = 0.0107), while no significant correlations were found between CD163 expression and the other clinicopathological parameters (Fig. 8G). However, for RNASE2 expression, no significant differences in any of the clinical pathological features mentioned above were observed. The above results indicated the expression of biomarkers can seldom be affected by clinicopathological features.

**Table 2:** Characteristics of the CD163 group participants

	Control	HF
*n*	20	20
Sex *(n male/n female)*	10/10	10/10
Age(years)	65.8 ± 10.9	73.2 ± 12.7
Smoke	5(25)	0(0)
Hypertension	12(60)	17(85)
DM	4(20)	3(15)
CAD	10(50)	13(65)
NT-proBNP(ng/ml)	112.84 ± 126.24	9562.20 ± 11,652.42
LDL-C(mmol/L)	2.21 ± 0.72	2.34 ± 1.47
cTnI(ng/ml)	0.050 ± 0.002	0.118 ± 0.160
eGFR(mL/min/1.73m^2^)	91.95 ± 11.55	59.25 ± 32.00

Data are mean ± SD or n (%). HF: Heart failure; DM: Diabetes mellitus; CAD: Coronary artery disease; NT-proBNP: N-terminal pro-B-type natriuretic peptide; LDL-C: Low-density lipoprotein cholesterol; cTnI: cardiac troponin I; eGFR: estimated glomerular filtration rate

**Table 3:** Characteristics of the RNASE2 group participants

	Control	HF
*n*	20	20
Sex *(n male/n female)*	9/11	11/9
Age(years)	67.2 ± 10.9	73.9 ± 12.8
Smoke	5(25)	0(0)
Hypertension	11(55)	16(80)
DM	3(15)	1(5)
CAD	10(50)	13(65)
NT-proBNP(ng/ml)	126.11 ± 126.92	5080.48 ± 10,380.75
LDL-C(mmol/L)	2.17 ± 0.63	2.53 ± 1.32
cTnI(ng/ml)	0.006 ± 0.002	0.244 ± 0.253
eGFR(mL/min/1.73m^2^)	91.95 ± 14.69	59.25 ± 27.78

Data are mean ± SD or n (%). HF: Heart failure; DM: Diabetes mellitus; CAD: Coronary artery disease; NT-proBNP: N-terminal pro-B-type natriuretic peptide; LDL-C: Low-density lipoprotein cholesterol; cTnI: cardiac troponin I; eGFR: estimated glomerular filtration rate

### Biomarker-specific transcription factors (TFs) and gene regulatory networks

**Fig. 9:**
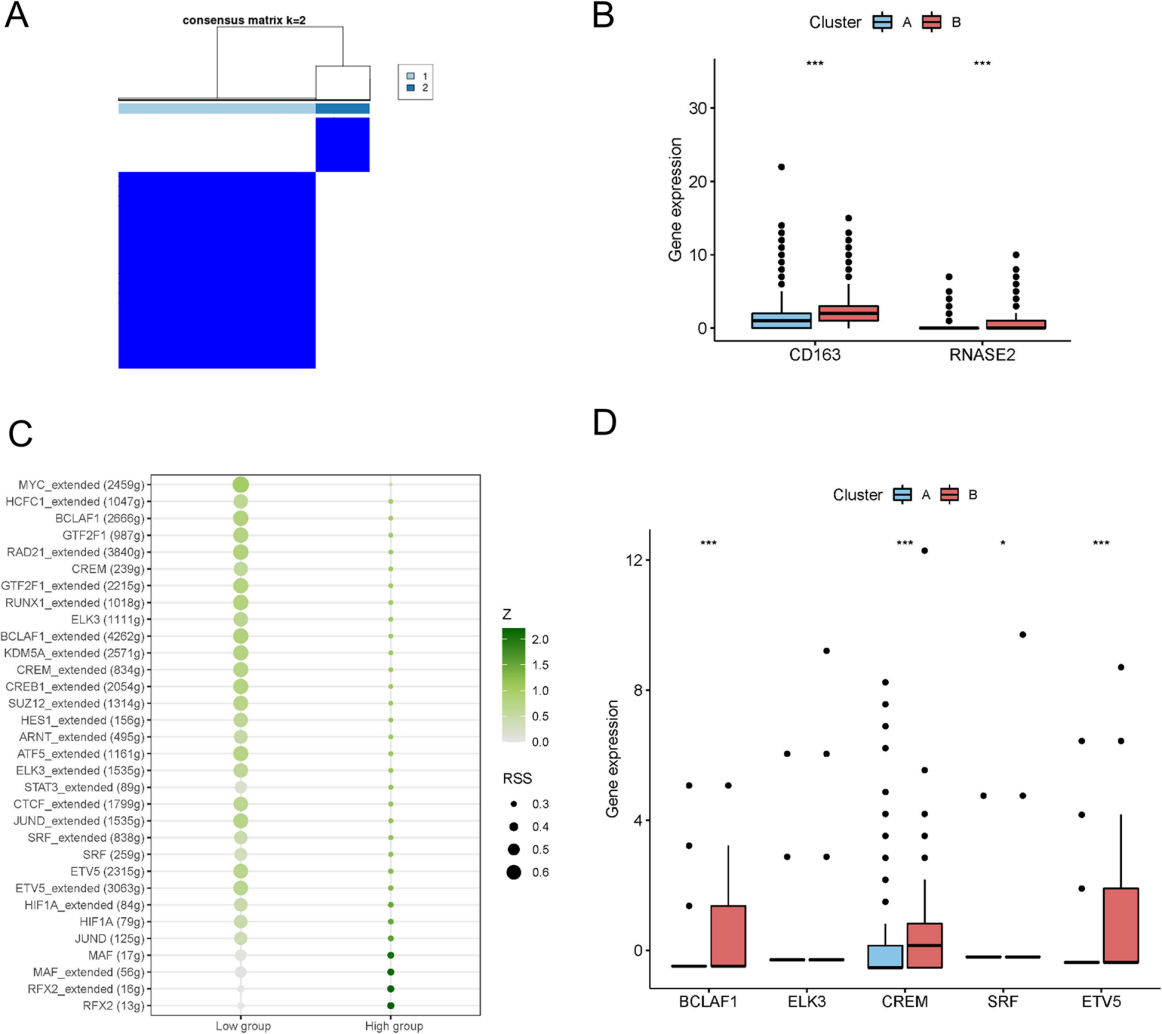
Biomarker-specific transcription factors and gene regulatory networks. (**A**) Consensus matrix plots depicting consensus values on a white to blue color scale ordered by consensus clustering when *k* = 2. (**B**) The expression levels of biomarkers in the two clusters. Statistical analysis was performed using the Wilcoxon rank sum test. (**C**) Dotplot showing transcriptional factors enriched in different clusters. (**D**) The expression profile of key TFs in two clusters. Statistical analysis was performed using the Wilcoxon rank sum test. Asterisks indicate significance, *p < 0.05; **p < 0.01; ***p < 0.001; ns, no statistical significance

Macrophage cell clusters were classified into 2 subtypes based on the expression of CD163 and RNASE2 (Fig. 9A, Supplementary Fig. 2B). Afterward, we plotted the expressions of biomarkers of the two clusters identified through consensus clustering, which revealed that the expression levels of CD163 and RNASE2 were remarkably higher in cluster B. Based on the biomarker expression levels, we labeled cluster A and cluster B as the low and high biomarker expression groups, respectively (Fig. 9B). Using SCENIC analysis, we also found that BCLAF1, GTF2F1, CREM, and ETV5 were specific motifs that had key roles in transcriptional regulation of the low group. HIF1A, MAF, and RFX2 motifs were activated in the high group (Fig. 9C). Moreover, we compared the expression of the top five TFs in the low biomarker group between the two expression groups. The results indicated that the expression of BCLAF1, CREM, and ETV5 were notably different between the two groups and hence they were identified as TFs that potentially could influence the expression of these biomarkers (Fig. 9D).

## Discussion

The clinical syndrome of HF can be caused by a variety of pathophysiologic changes, such as myocardial ischemia and infarction, pressure or volume overload, and responses to viral infections. Irrespective of the underlying etiology, excessive, uncontrolled, or dysregulated inflammation can worsen myocardial injury, which in turn can contribute to the advancement of HF [23]. Immune cells are essential in the inflammatory process and are believed to modulate HF progression. During the past three decades, experimental and clinical studies have enhanced the comprehension of the involvement of inflammation and immune cells in the development of HF. Although there were initial setbacks in translating clinical treatments, targeting the interactions between inflammation and immune cells remains a promising and appealing direction for HF treatment [24]. Recently, machine learning has been leveraged for screening, diagnosis, and prognosis of diseases such as the prediction of cardiovascular events [25], detection of colorectal cancer [26], diagnosis of childhood B-cell acute lymphoblastic leukemia [27], and prediction of non-small cell lung cancer [28]. Currently, blood biomarkers such as BNP, NT-proBNP, cTn, Galectin-3, Soluble ST2(sST2), and Growth differentiation factors-15(GDF-15) [29, 30] are used to predict and diagnose HF [31]. While BNP and NT-proBNP are widely regarded as the gold standard in prognostic diagnosis and stratification of HF, their sensitivity in the diagnosis of acute HF and Heart failure with preserved ejection fraction (HFpEF) may be low [32, 33], and their accuracy is influenced by various factors, including age, sex, ethnicity, genetic variants, and numerous cardiac and non-cardiac conditions [34]. Novel biomarkers, such as sST2, GDF-15, and Galectin-3, show promise in evaluating prognosis beyond known natriuretic peptides. However, their role in the clinical management of patients is not well defined, and further research is necessary [35]. Regrettably, the prognosis of patients with HF remains poor as a result of insufficient early diagnosis and effective treatment options.

Here, we obtained bulk data on HF, utilized deconvolution techniques, and analyzed the discrepancies in immune cells between HF and normal samples. The proportion of immune cells was observed with a significant difference between HF and normal, such as macrophages, neutrophils, and mast cells, suggesting that dysregulation of the immune microenvironment is the main reason for the progression of HF. We further explored the key cluster (macrophages) in HF by bioinformatics analysis and machine learning. We distinguished differences in macrophage composition between HF and normal through annotation and cluster analysis of single-cell data, which may indirectly lead to differences in biological processes between HF and normal. Additionally, we conducted differentiation trajectory analysis and pseudo-time analysis of macrophages to identify various differentiation states of macrophages. The macrophages in patients with HF exhibited five states and are related to multiple metabolic processes such as respiratory electron transport chain and oxidative phosphorylation in the procession of differentiation. An imbalance of macrophage polarization between pro-inflammatory M1 and anti-inflammatory M2 phenotypes can lead to excessive inflammation and cardiac injury, ultimately resulting in HF. This dysregulation is associated with metabolic rearrangement between glycolysis and mitochondrial oxidative phosphorylation that influences macrophage polarization. Therefore, factors that impact macrophage metabolism have the potential to disrupt the balance between M1 and M2 phenotypes and aggravate inflammation [36]. According to this evidence, our analysis findings are highly precise and credible, suggesting that advocate for the exploration of the underlying molecular mechanisms through the identification of macrophage energy metabolism.

Moreover, we further explored the interaction between macrophages and other cells. Through the analysis of cellular communication in our research, we have identified multiple reliable ligand-receptor pairs that have facilitated our understanding of the regulatory network within the immune microenvironment of HF. Macrophages express major receptors of the IL-16 signaling pathway and influencers of the CCL signaling pathway, which aligns with previous research. CCL2 plays a crucial role in adverse remodeling, fibrosis, and dysfunction in patients with both infarctive and non-infarctive HF [37, 38] and has been proposed as a potential therapeutic target for conditions related to myocardial injury and adverse remodeling [39]. Previous studies also revealed that elevated expression of IL-16 within the heart leads to increased cardiac fibrosis and left ventricular myocardial stiffening, which is accompanied by infiltration of macrophages [40].

Next, we extracted MDRGs that are essential in macrophage differentiation trajectories. Based on macrophage-related modular genes, MDRGs, and DEGs between HF and normal samples, we developed a risk model to predict prognosis that is composed of two hub genes (CD163 and RNASE2). CD163 is a receptor expressed by monocytes/macrophages, and the shed soluble CD163(sCD163) reflects monocyte/macrophage activation, which plays a critical role in mediation of chronic inflammatory activation in HF [41, 42]. RNASE2 is a cytotoxic protein secreted mainly by eosinophils and macrophages, and it has antiviral and chemotactic activities in vitro [43, 44]. Yang et al. showed that RNASE2 is capable of activating human dendritic cells, resulting in the production of multiple inflammatory cytokines, growth factors, chemokines, and soluble receptors. In addition, RNASE2 was found to be capable of inducing the maturation of dendritic cells [45]. By using clinical samples, we validated our results, which contributed to the reliability and accuracy of our findings. Utilizing RT-qPCR to evaluate the expression levels of the two hub genes in blood samples, we determined that these genes have the potential to distinguish between HF and non-HF individuals. The study observed that CD163 and RNASE2 were significantly downregulated in patients with HF when compared to the normal samples, indicating that these two genes may have a protective effect against the advancement of HF. Together, CD163 and RNASE2 were considered as candidate biomarkers of HF. Moreover, our results indicated a strong association between CD163 and hypertension, consistent with results from previous studies [46]. A prior study provided evidence that the level of CD163 expressed on monocytes in individuals with coronary heart disease exhibited a positive correlation with low-density lipoprotein cholesterol [47]. However, this difference was not shown in our experiment, probably due to the fact that our sample size was relatively small. In a previous study, it was demonstrated that sCD163 levels in plasma were indicative of the complete pool of membrane-bound CD163 [48]. Conversely, another study found that there was a negative relationship between the expression of CD163 on the surface of monocytes and the concentration of sCD163 [49]. The inconsistent outcomes may be attributed to differences in the patient cohorts investigated. The former study was conducted in infected hematologic patients, which showed elevated sCD163 due to increased CD163-expressing macrophages or upregulation of CD163 gene expression by pro-inflammatory mediators, whereas the other study was conducted in randomized subjects. In our investigation, the expression level of sCD163 in peripheral blood may be consistent with CD163 in myocardial tissue. Moreover, the expression level of CD163 was found to be positively correlated with eGFR in patients with HF. Patients with ANCA-associated glomerulonephritis were also found to exhibit significantly raised levels of U-sCD163 [50]. Our findings showed a negative correlation between NT-proBNP and RNASE2. This suggested that RNASE2 has the potential to be used in combination with NT-proBNP for the diagnosis of HF. In addition, we analyzed the diagnostic values of both hub genes in our cohort using ROC curve analysis. Both genes had reliable diagnostic values, exhibiting remarkable specificity and sensitivity. Taken together, the evidence presented above suggests that CD163 and RNASE2 can serve as distinct factors and diagnostic indicators for HF.

We acknowledge several limitations in this study. First, the trial involved a limited sample size and restricted patient inclusion for clinical characteristics, which might result in biased outcomes. Second, multiple datasets with different control numbers would affect the interpretation of the findings. Next, we plan to continue collecting cases for a multicenter, large-sample study to confirm our findings. Further research is required to investigate the role of CD163 and RNASE2 in the development of HF in vivo and in vitro. Despite these limitations, our study provides valuable insights into the specific macrophage-associated biomarkers that could enable the rapid diagnosis of patients with HF. These findings offer novel insights into the prevention and treatment of HF and could potentially serve as a basis for future investigations.

## Conclusion

The key role of macrophages in HF was screened by machine learning and a logistic regression diagnostic model based on macrophage-related genes was constructed. The diagnosis model was evaluated and confirmed by bioinformatic analysis and experiments, which may contribute to a new perspective for the prevention and treatment of HF and provide a basis for follow-up research.

### Supplementary Information

**Additional file 1.**

**Additional file 2.**

## Abbreviations

HF: Heart failure
DEGs: Differentially expression genes
MDRGs: Macrophage differentiation-related genes
GO: Gene annotation enrichment analysis of gene ontology
ROC: Receiver operating characteristic
AUC: The area under the ROC curve
BPs: Biological processes
CCs: Cellular components
MFs: Molecular functions
UMAP: Unified modal approximation and projection
DC: Dendritic cells
PCA: Principal components analysis
HFpEF: Heart failure with preserved ejection fraction
LDL-C: Low-density lipoprotein cholesterol
DM: Diabetes mellitus
NT-proBNP: N-terminal pro-B-type natriuretic peptide
cTnI: Cardiac troponin I
eGFR: Estimated glomerular filtration rate
