# Supplementary material for: Integrating scRNA-seq to explore novel macrophage infiltration-associated biomarkers for diagnosis of heart failure
Source: BMC Cardiovasc Disord. 2023 Nov 16;23:560. doi: 10.1186/s12872-023-03593-1 (PMC10652463; doi:10.1186/s12872-023-03593-1)
Supplement: Supplementary file 2 — Additional file 2. [file 12872_2023_3593_MOESM2_ESM.pdf]

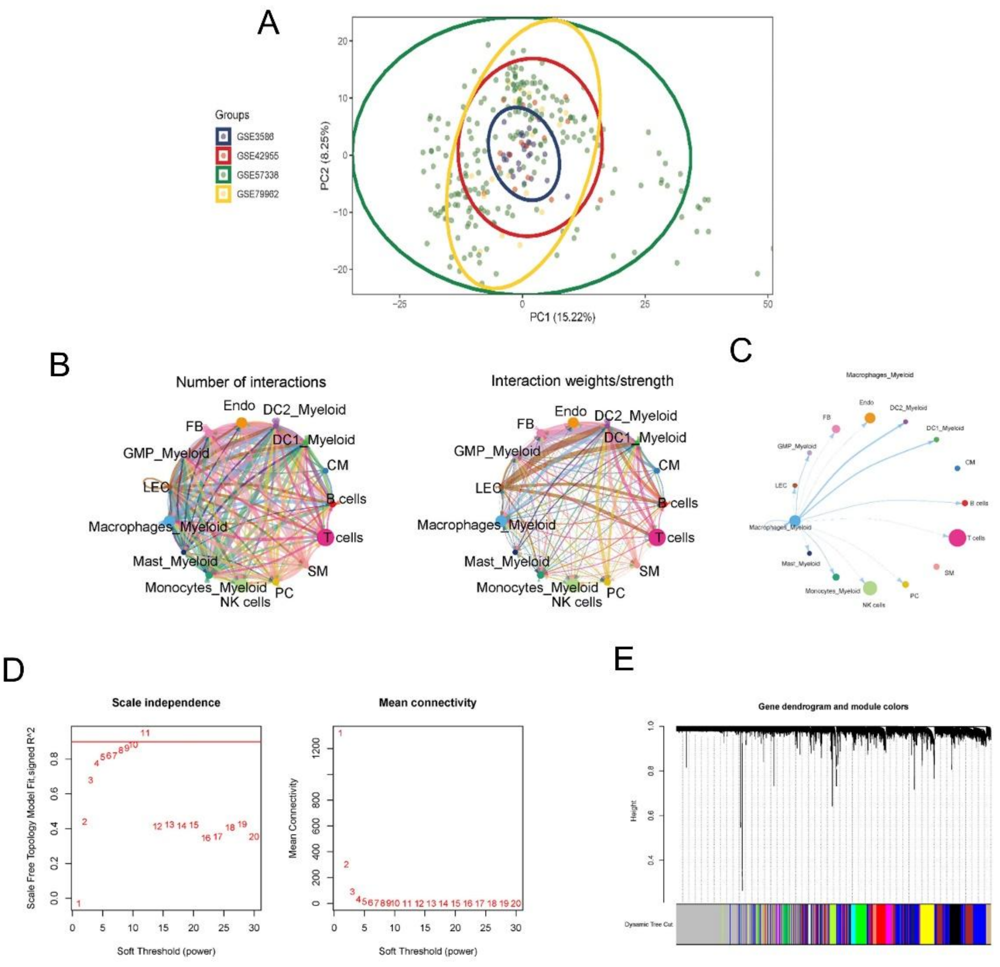

Supplementary Figure 1. (A) Principal component analyses of expression profile after batch effect correction. (B)An overview of cell-cell interactions. Arrow and edge color indicate direction. Edge thickness indicates the number (Left) or the weighted (Right) of interaction between populations. The loops indicate autocrine circuits. (C) Communication between macrophages and other cells. (D) Scale-free co-expression network and Soft threshold and average connectivity value. (E) Dendrogram of all differentially expressed genes clustered.

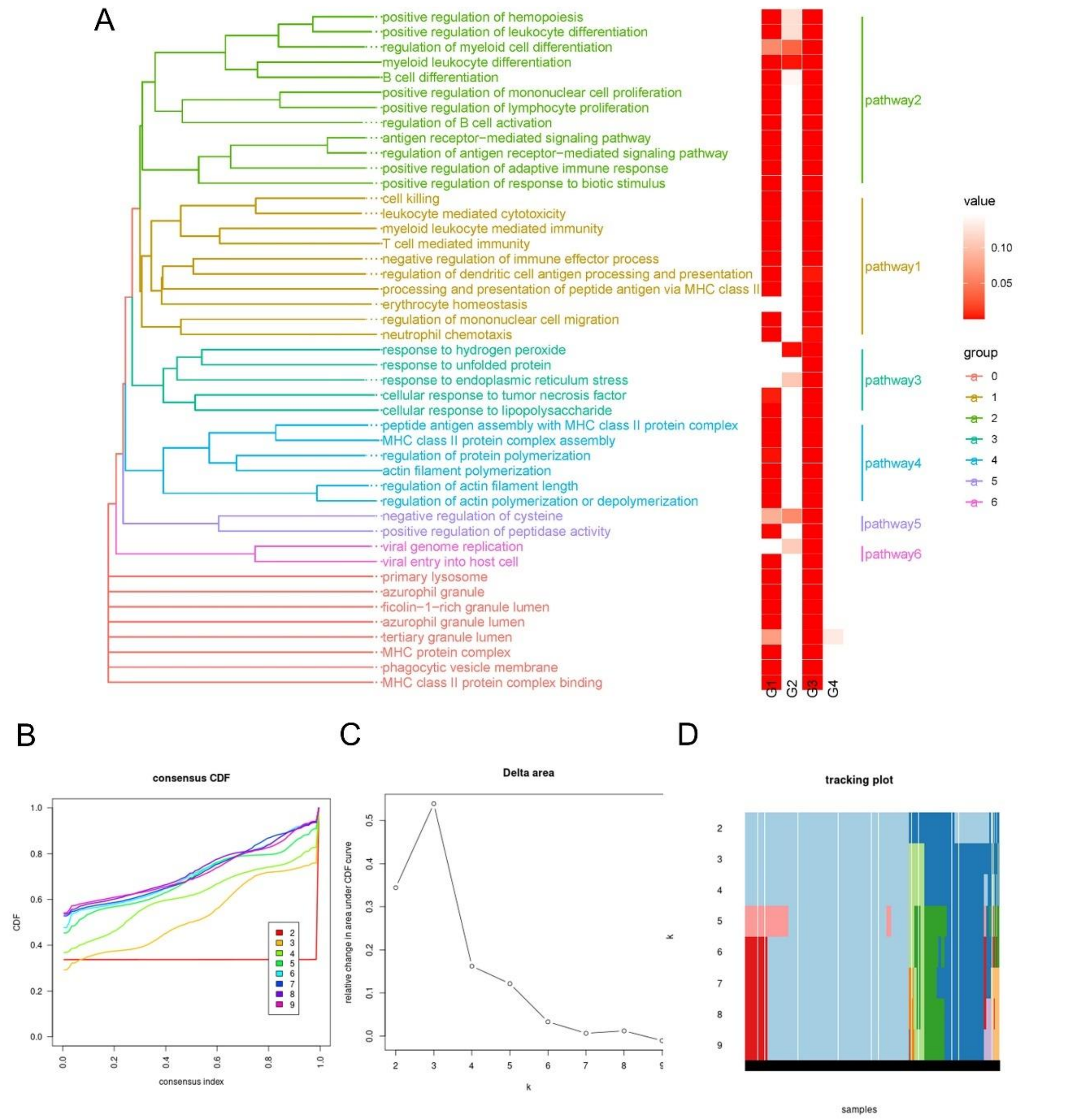

Supplementary Figure 2. (A) Go analysis of genesets. (G1: genes in black module; G2: genes in yellow mudules; G3: MDRGs; G4: DEGs between HF and normal) (B) Empirical CDF plots displaying consensus distribution. (C) Relative change in the area under the CDF curve for k = 2-9. (D) An item tracking plot showing the consensus cluster of items (in columns) at each k (in rows).
